# Amniotic Membrane Patch Graft in Management of Double Chamber after Deep Anterior Lamellar Keratoplasty

**DOI:** 10.18502/jovr.v15i4.7795

**Published:** 2020-10-25

**Authors:** Mehran Zarei-Ghanavati, Mahmood Davoodabadi, Ahad Shahbazi

**Affiliations:** ^1^Translational Ophthalmology Research Center, Farabi Eye Hospital, Tehran University of Medical Sciences, Tehran, Iran

**Keywords:** Amniotic Membrane, DALK, Descemet Perforation, Double Chamber

## Abstract

**Purpose:**

To describe a novel technique of amniotic membrane (AM) patch graft in the management of double chamber treatment after big-bubble deep anterior lamellar keratoplasty (DALK).

**Case Report:**

A 35-year-old male patient with advanced keratoconus underwent big-bubble DALK. Manual lamellar dissection was done due to failed big-bubble. First-day postoperative double chamber was detected. Air bubbling and SF6 injection were tried without any success. Double chamber resolved by fixation of AM transplantation patch graft (1 × 1 mm) over the Descemet's membrane perforation with fibrin glue.

**Conclusion:**

Amniotic membrane patch graft can be used in the management of double chamber after DALK not responsive to intracameral gas injection.

##  INTRODUCTION

Deep anterior lamellar keratoplasty (DALK) is a preferred transplantation technique for advanced keratoconus. Higher graft survival is the main advantage of DALK over penetrating keratoplasty.^[1]^ Big-bubble and Melles technique are common methods for DALK. One of the major complication of these techniques is iatrogenic Descemet' membrane (DM) perforation.^[1]^ This complication may occur during all stages of DALK from trephination to graft suturing. The incidence of DM perforation during DALK was reported up to 39%.^[2]^ DM perforation may cause double anterior chamber between donor stroma and recipient DM.

Although DM may be reattached spontaneously with a good visual outcome in some cases,^[3,4]^ surgical management of DM detachment is often necessary. It includes injection of air or gas into the anterior chamber, suturing DM to its position, or even repeating keratoplasty.^[5,6,7]^

**Figure 1:**
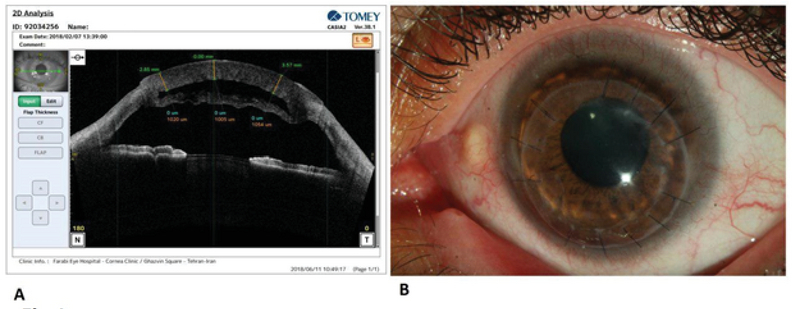
Management of a DM perforation in a DALK case. (A) Anterior segment-OCT (AS-OCT) image showing a double anterior chamber and detached Descemet membrane (double chamber was not resolved after two attempts of gas injection into the anterior chamber). (B) One week after the surgery: Slit lamp photograph shows clear corneal graft with resolved double chamber. Amniotic membrane patch graft is detectable between donor and recipient at the site of Descemet perforation.

We report a novel technique of using amniotic membrane (AM) as a patch for repairing the site of DM perforation refractory to anterior chamber gas injections.

##  CASE REPORT

A 35-year-old male patient diagnosed with advanced KCN was referred to us with. He had a complaint of gradually decreased vision in the left eye with the best-corrected visual acuity (BCVA) of 2/10. Rigid gas permeable lens could not be fitted for the patient. The left eye had the maximum keratometry of 75.4 diopters and minimum pachymetry of 397 microns. The patient underwent DALK with big-bubble technique. After partial trephination of 8 mm diameter, 27-gaugue needle attached to a 5 ml syringe of air was advanced from the superior rim of trephination toward paracentral zone of the cornea. During the air bubble expansion, DM was ruptured, and air penetrated to the anterior chamber (AC). A tear was detected in the inferior nasal periphery of DM in a diameter of nearly 0.3 mm. Deep stromal dissection was achieved with Melles' spatula and 80% air was injected in the AC at the end of operation.

Double chamber was seen on the first day postoperatively. There was no change in size of double chamber after 4 days of medical treatment. Therefore, injections of intracameral air and then SF6 were done without any success.

We decided to patch DM perforation site with AM graft. Under general anesthesia, all donor graft sutures were removed and graft was put in normal saline container. Air was injected in the AC and DM was dried with sponge. A patch (1 × 1 mm) of cryopreserved acellular AM (SinaCell, Iran) was fixed over the perforation site with small amount of fibrin glue. The same graft was sutured to the receipt rim with 10-0 nylon separate sutures.

On the first postoperative day, mild diffuse cornea stromal edema without noticeable double chamber was present. At one week after surgery, cornea was clear with the donor attached to DM without double chamber (Figure 1). Fifteen months after the surgery, BCVA with –4.5–4.5 × 65 was 6/10.

##  DISCUSSION

This case showed that grafting of AM patch is an effective procedure in post-DALK double chamber after failure of air bubbling. AM has been used as a biological membrane for various indications including chemical burns, persistent epithelial defects, ulcers, and pterygium surgery. Many studies have shown AM's clinical efficacy to stimulate wound healing, promoting epithelization, while suppressing inflammation, angiogenesis, and scarring. This tissue is used in two ways: (1) inlay technique that is applied as a permanent basement membrane substitute and (2) onlay technique that is temporarily placed on the ocular surface.^[8,9]^ In this case, we used AM as a patch to seal the DM perforation site as a new indication for AM.

Although, the literature suggests air injection for the management of double chamber, some studies have reported that the air or gas bubble can cause pupillary block with a fixed dilated pupil (Urrets–Zavalia syndrome), anterior subcapsular lens opacities, and endothelial cell loss more than 20%.^[10]^ Furthermore, tamponade of inferior DM perforation with gas is a challenging issue. The use of fibrin glue application was successful in sealing the DM perforation.^[11]^ Another report described that stromal patch graft can be used in combination with fibrin glue after the failure of glue application alone.^[12]^

In our case, we observed perforation of DM at inferonasal area during big-bubble technique. The aqueous then penetrated the space between DM and graft stroma. There was persistent double chamber even after air and SF6 injection. Only after AM patch graft, the perforation site was closed and double chamber resolved. AM will not prevent from good apposition of DM and posterior surface of cornea because it is very thin and smooth in comparison to stromal patch. Moreover, it will remain longer than fibrin glue alone to stop any leakage from the perforation site and increase the success rate. However, there are some limitations for the application of AM patch in sealing of the DM perforation. Some or all sutures should be removed to put AM over the perforation site and glue it. We also observed interface haziness due to AM remaining until the last follow-up (15 months). Therefore, AM patch cannot be used if the DM perforation site is located in the optical zone.

In summary, it seems that the application of AM patch to seal non-central perforations of DM is helpful to manage double chamber after DALK not responsive to air bubbling.

## References

[1] Borderie VM, Sandali O, Bullet J, Gaujoux T, Touzeau O, Laroche L. Long-term results of deep anterior lamellar versus penetrating keratoplasty. *Ophthalmology* 2012;119:249–255. 10.1016/j.ophtha.2011.07.057 22054997

[2] Sugita J, Kondo J. Deep lamellar keratoplasty with complete removal of pathological stroma for vision improvement. *Br J Ophthalmol* 1997;81:184–188. 10.1136/bjo.81.3.184 PMC1722147 9135380

[3] Passani A, Sframeli AT, Loiudice P, Nardi M. Late spontaneous resolution of a double anterior chamber post deep anterior lamellar keratoplasty. *Saudi J Ophthalmol* 2017;31:58–60. 10.1016/j.sjopt.2017.01.003 PMC5352942 28337067

[4] Iradier MT, Moreno E, Aranguez C, Cuevas J, García Feijoo J, Garcia Sanchez J. Late spontaneous resolution of a massive detachment of Descemet's membrane after phacoemulsification. *J Cataract Refract Surg* 2002;28:1071–1073. 10.1016/s0886-3350(01)01220-2 12036658

[5] Chow VW, Agarwal T, Vajpayee RB, Jhanji V. Update on diagnosis and management of Descemet's membrane detachment. *Curr Opin Ophthalmol* 2013;24:356–361. 10.1097/ICU.0b013e3283622873 23665525

[6] Assia EI, Levkovich-Verbin H, Blumenthal M. Management of Descemet's membrane detachment. *J Cataract Refract Surg* 1995;21:714–717. 10.1016/s0886-3350(13)80573-1 8551454

[7] Kumar H, Ali MS, Mishra D. Management of Descemet's membrane detachment by intra cameral air injection. *Ann Int Med Den Res* 2016;2:OT01–OT04.

[8] Clare G, Suleman H, Bunce C, Dua H. Amniotic membrane transplantation for acute ocular burns. *Cochrane Database Syst Rev* 2012;9:CD009379. 10.1002/14651858.CD009379.pub2 PMC8966384 22972141

[9] Rahman I, Said DG, Maharajan VS, Dua HS. Amniotic membrane in ophthalmology: indication and limitations. *Eye* 2009;23:1954–1961. 10.1038/eye.2008.410 19169225

[10] Maurino V, Allan BD, Stevens JD, Tuft SJ. Fixed dilated pupil (Urrets-Zavalia syndrome) after air/gas injection after deep lamellar keratoplasty for keratoconu*s. Am J Ophthalmol* 2002;133:266–268. 10.1016/s0002-9394(01)01308-3 11812433

[11] Anwar HM, El-Danasoury A, Hashem AN. The use of fibrin glue to seal Descemet membrane microperforations occurring during deep anterior lamellar keratoplasty. *Cornea* 2012;31:1193–1196. 10.1097/ICO.0b013e318242fd94 22495028

[12] Ghaffari R, Ghassemi H, Latifi G, Jabbarvand M, Zamzam A, Hashemi H. Stromal Patch with fibrin glue as a novel surgical technique to seal peripheral Descemet's membrane perforations in deep anterior lamellar keratoplasty. *Int Ophthalmol* 2019;39:2275–2282. 10.1007/s10792-018-01065-6 30656510

